# On the Mode I and Mode II Delamination Characteristics and Surface Morphological Aspects of Composites with Carbon-Thermoplastic Hybrid Fabrics and Innovative Liquid Thermoplastic Resin

**DOI:** 10.3390/polym14194155

**Published:** 2022-10-04

**Authors:** Somen K. Bhudolia, Goram Gohel, Durga Vasudevan, Kah Fai Leong, Pierre Gerard

**Affiliations:** 1 School of Mechanical and Aerospace Engineering, Nanyang Technological University, 50, Nanyang Avenue, Singapore 639798, Singapore; 2 School of Mechanical and Aerospace Engineering, Technical University of Munich Asia, 25 International Business Park Rd, Singapore 609916, Singapore; 3 Groupement de Recherche de Lacq, Arkema, Route Départementale 817, BP 34, 64170 Lacq, France

**Keywords:** hybrid fibers, hybrid composites, thermoplastic resin, vacuum assisted resin infusion (VARI), fracture toughness, fractography

## Abstract

In the current research, the delamination behavior under Mode I and Mode II loading for the hybrid carbon-thermoplastic fabrics in conjunction with novel liquid thermoplastic acrylic Elium^®^ resin processable at ambient conditions was studied. The experimentation by incorporating doublers methodology, studying the performance under Mode I and Mode II loading, and understanding failure mechanisms using surface morphological fractography is deliberated. Hybrid Carbon-Ultra-high molecular weight polyethylene (UHMWPP)/Elium^®^ composite has shown a 22.81% higher G_IC_ and a 22.2% higher G_IIC_ than Carbon-UHMWPP/Epoxy composite. On the contrary, the Carbon_Ultra-high molecular weight polypropylene (UHMWPE)/Elium^®^ has shown an 11.11% higher Mode I critical energy release rate (G_IC_) and a 7.58% higher Mode II critical energy release rate (G_IIC_) than Carbon_UHMWPE/Epoxy composite. Hybrid fiber reinforced thermoplastic composites have shown severe plastic deformation of the matrix, rough fracture surface, and micro-cracks on the de-bonding surface, extensive fiber bridging, and crack branching which contributed to the improvement in the delamination behavior. Hybrid fiber architecture is also found to be detrimental by inducing crack arresting mechanisms including the tortuous crack path and the resin-rich pockets path due to the mismatch of the size of the fiber yarns.

## 1. Introduction

Composite materials with carbon as a reinforcement material are significantly used in many industries in wide applications ranging from automotive, aerospace, marine and offshore, and many others as they have lightweight characteristics along with high specific mechanical properties, such as strength and stiffness. Although considered by many as a wonder material along with the obsession of industries to use them, there occurs a challenge in terms of cost along with their brittle nature which leads to catastrophic failure with low strain to failure under different loading scenarios. While the polymeric fibers including ultra-high molecular weight polyethylene (UHMWPP), ultra-high molecular weight polypropylene (UHMWPE), Polyester (Diolen) are ultra-lightweight in nature, have excellent impact and toughness characteristics, and have a high elongation to break, and consequently are appealing for sporting goods, body armors, and ballistic applications [1,2]. However, they also have certain limitations with extremely low shear and compressive strength and hence are not ideal for structural load carrying applications. More recently, the hybridization route has been explored by many textile industries, such as DSM, Innegra Tech and others, to manufacture hybrid thermoplastic fabrics in conjunction with carbon fiber which could mitigate the effect of brittleness and bring in ductility to the reinforcement system while simultaneously improving the in-plane compressive and shear properties as well [3,4]. If effectively used, the hybridization technique could result in optimized properties as there is a greater degree of design space using this route.

Woven ultra-high molecular weight polypropylene (UHMWPP), commercially known as Innegra™, is a thermoplastic fiber made from polyolefin polypropylene. Innegra™ is a very lightweight fiber, with a density of 0.84 g/cm^3^ and a high elongation to break of 9.5%, when compared to other commonly used fibers in fiber reinforced polymer (FRP) laminate composites. It is also non-electrical and hydrophobic, due to its limited moisture absorption property. However, because of its low tensile strength of 667 MPa and modulus of 15 GPa when compared to other composite fibers, Innegra™ is frequently employed in hybrid composites, which are made up of two or more distinct fibers that are reinforced in a resin matrix. Various other characteristics of Innegra™ include high toughness, high crystallinity, and excellent impact properties. Innegra™ is frequently utilized in a hybrid fiber system, such as a carbon fiber/UHMWPP hybrid weave because of their low modulus. For specific applications, this combines the desirable qualities of both fibers, namely, carbon fiber’s high flexural modulus and Innegra’s high impact resistance. Innegra™ fiber reinforced composites are generally used in kayaks, ice-hockey sticks, helmets, and many more. DSM’s Dyneema^®^ fiber is an extremely strong ultra-high molecular weight polyethylene (UHMWPE) fiber that provides maximum strength with less weight [5]. As a result, the number of viable applications is practically limitless. Dyneema^®^ fiber is made using a gel spinning process that involves drawing, heating, elongating, and cooling the fibers. Low density, high crystallization, and molecular alignment are the results of stretching and spinning. Extremely long molecular chains found in Dyneema^®^ transfer load more efficiently to the polymer backbone. As a result, Dyneema^®^ outperforms competing materials in terms of strength while also being lighter. In the fiber direction, Dyneema^®^ has high strength and modulus (resistance to deformation). Because of its low density, it has an incredibly high strength per unit of weight, making it one of the strongest man-made fibers. The elongation at break is modest, but the energy to break is high due to the high strength. The mechanical properties of this synthetic fiber are unaffected by the presence of water, unlike those of conventional synthetic fibers. The modulus and strength in the transverse direction are lower than in the fiber direction due to the anisotropic nature of the fibers. Dyneema^®^ finds its application in various products, such as helmets, soft and hard armors, and anchor ropes. Generally, Dyneema^®^ fibers are also hybridized with carbon fibers to achieve the desired properties of both the fibers.

Using the thermoplastic polymer fabrics with thermoplastic resin can potentially lead to wholly thermoplastic structures and even the usage of hybrid thermoplastics can keep the overall proportion of thermoplastic presence higher compared to that with pure carbon or with a thermosetting matrix variant. High-performance thermoplastic resins, such as PEEK, CBT, and PU are extensively used to manufacture composite laminates [6,7,8]. Yet, the liquid injection processes can be employed directly with these matrices as they are accessible in pellet or film form and in turn must be processed at high-temperature ranges [9]. An acrylic thermoplastic Elium^®^ was recently developed by ARKEMA [10,11,12,13,14,15,16,17,18] to address the challenges associated with manufacturing with the potential to cure at room temperature. Elium^®^ composites have been shown to have superior fracture toughness, impact resistance, and vibration damping qualities [11,16,19] compared to the composites with thermosetting epoxy as matrix material. Fracture toughness [9,11,20], vibration [19,21], flexural properties [13,21,22], tensile properties [18,23], weldability [21] and other mechanical properties for acrylic-based composites were all comprehensively researched.

For any material system to be readily adopted by industries, it is of paramount importance to understand the bonding characteristics of the fabrics and the matrix system. If there is good compatibility, the structure will carry a significant load otherwise there significant delamination will occur. The delamination phenomenon initiates due to poor interfacial adhesion during Mode I (peel mode), Mode II (shear mode), and Mode III (torsion mode) type of loading scenarios [24,25]. There is limited work on understanding the bonding characteristics of a thermoplastic matrix with only the bonding capability of thermoplastic Elium^®^ resin with UHMWPE fabrics studied by Shanmugam et al. [20]. They have determined that UHMWPE fabrics have relatively weak bonding with Elium^®^ matrix and the reason is the lack of the polar functional group on the fabrics [26]. Whereas in another study the surface treated UHMWPE fabric improved the bonding characteristics. In Mode I fracture toughness tests, Somen K. Bhudolia et al. [9] observed that thin ply Elium^®^ composite had a 30% greater ILFT than thick ply Elium^®^ composite. In addition, thin Elium^®^ composites have a 72% improvement in ILFT when compared to thin Epoxy composites. Logesh Shanmugam et al. [20] researched enhancing the fracture toughness in Mode I of Elium^®^ composite by enhancing the fiber/matrix bonding with the help of surface treatments. This resulted in an improvement in G_IC_ by 19.6% and 42.5% for the PDA surface-treated sample and PDA with 0.03 wt% of MWCNT ingrained, respectively, compared to non-treated composite laminate. A detailed study was carried out by L.C.M. Barbosa et al. [11] regarding the fracture toughness in Mode II of Elium^®^-based composites. In this study, for composite laminate a reinforcement carbon fiber along with thermoplastic Elium resin and thermoset Epoxy resin were used. Mode II fracture toughness of Carbon/Elium^®^ composites was obtained to be 214.22 J/m^2^, which is 40% higher compared to the Carbon/Epoxy composites.

Concerning the hybrid composites with hybrid fabrics (carbon + thermoplastic fabrics), the majority of the work which has been reported in the literature is concentrated on studying the tensile, fatigue, impact, and damping characteristics of these hybrid composite structures [2,18,27,28,29,30,31,32]. However, there is very limited research on understanding the fracture toughness attributes of composites with hybrid thermoplastic fabrics. Recently, Zhao et al. studied the Mode I and Mode II attributes of the carbon-Dyneema hybrid composites with thermosetting epoxy resin as the matrix material and concluded the usage of Dyneema could potentially act as a toughening mechanism to improve delamination resistance characteristics. They have shown that using the hybridization of carbon fabrics with Dyneema fabrics can potentially increase the Mode I and Mode II performance by 65% and 40%, respectively, compared to that with pure carbon [3]. In another study by Zou et al., the performance of hybrid carbon-Dyneema composites was studied with an emphasis on understanding the details of failure mechanisms governing the increase in the Mode II properties due to hybridization [4]. In addition, they have shown that the J-integral technique and digital image co-relation techniques lead to more consistent fracture toughness results.

After a detailed literature review and addressing the research gaps, there is no research reported on understanding the fracture toughness attributes in Mode I and Mode II of hybrid thermoplastic composites with acrylic Elium^®^ resin. In the current investigation, authors have researched the characteristics, in Mode I and II, of the composites manufactured with the hybrid fabrics (carbon + UHMWPP and carbon + UHMWPE) and using novel acrylic resin, Elium^®^ as the matrix material to understand their adhesion characteristics. Current work also utilizes a testing methodology using doublers to avoid the large displacement and compression failure constraints. Failure mechanisms are also studied in detail under electron microscopy and SEM and the comparisons are performed with a thermosetting epoxy variant. The usage of these hybrid thermoplastic fabrics with thermoplastic Elium^®^ resin could potentially further improve the impact and damping characteristics and could be a tremendous material system for automotive, sporting goods, helmets, bicycle frames, and lightweight armor applications. Hence, the current investigation is an important step to understanding the delamination behavior of these fabrics with acrylic Elium^®^ resin and accessing their suitability to be used for the above-mentioned applications.

## 2. Materials and Manufacturing

### 2.1. Materials

**Figure 1:**
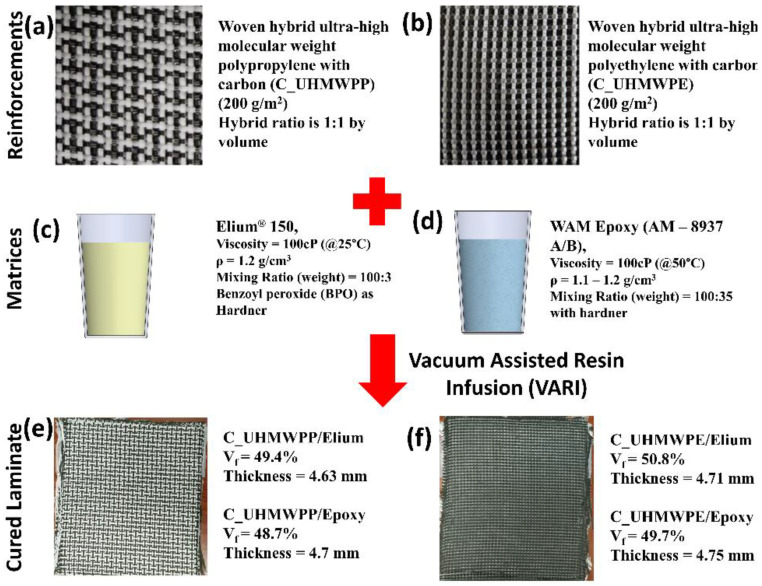
Properties of different fibers, resin system and laminates (**a**,**b**) C_UHMWPP and C_UHMWPE reinforcement system (**c**,**d**) Elium 150 and Epoxy matrix system (**e**,**f**) C_UHMWPP/Elium and C_UHMWPE/Elium cured laminates.

In this current research, two hybrid configurations of thermoplastic fibers, UHMWPP and UHMWPE with carbons were manufactured and investigated (refer Figure 1). It is to be noted that the ratio of the thermoplastic fiber to the carbon fabrics in hybrid configurations is 1:1. The densities of the hybrid UHMWPP and Hybrid UHMWPE reinforcements are 1.31 g/cm^3^ and 1.38 g/cm^3^.

Thermoplastic and thermoset matrices were used in the current project along with hybrid reinforcement for manufacturing the composite panels. A liquid thermoplastic resin at room temperature (RT) Elium^®^ 150, from Arkema, France with a viscosity of 100 cP at RT was used as a thermoplastic variant [9,16,33,34]. On the other hand, Epoxy (AM-8937 A/B) resin, procured from Wells Advanced Materials Co., Ltd., Shanghai, China, is employed as a thermoset variant for composite manufacturing. For creating an artificial crack into the manufactured laminate Polytetrafluoroethylene (PTFE) film of 25.4 μm thickness was used for Mode I and 12.5 μm thickness was used for Mode II. The adhesive utilized for the gluing of the composite laminates to aluminum doublers is Bostik’s SAF 30 ultimate M10 grade and aluminum 6065 sheets with a thickness of 1.2 mm were used as doublers for the fracture toughness tests—Mode I and Mode II.

### 2.2. Manufacturing

**Figure 2:**
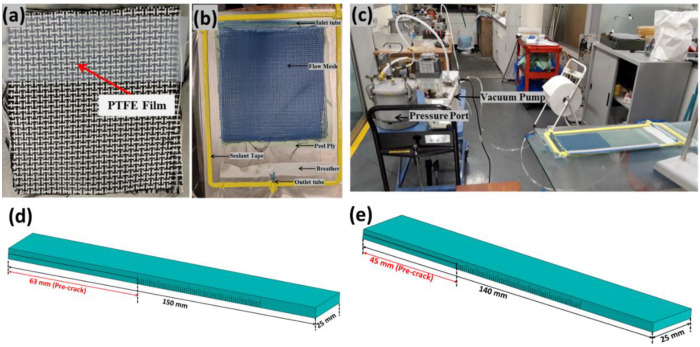
(**a**) Placement of PTFE film inserted in the central layer of the preform (**b**,**c**) Resin Infusion setup (**d**) Manufactured laminate cut to the dimension for Mode I (**e**) Manufactured laminate cut to the dimension for Mode II.

For the fracture toughness tests, the hybrid thermoplastic and thermoset composites were fabricated using the conventional vacuum-assisted resin infusion (VARI) manufacturing process, as illustrated in Figure 2. PTFE film was inserted in the central layer of the dry hybrid fabric preform as shown in Figure 2a.

Before manufacturing, the mould was prepared by applying multiple layers of the mould releasing agent. Then, the fibers were laid on a mould; inlet, and outlet tubes were connected; and the VARI setup was prepared as depicted in Figure 2b,c. Lastly, the entire set-up was sealed using sealant and a vacuum bag and a leak test was performed. Once the leak test was cleared the setup was ready for infusion. It is to be noted that 12 layers of hybrid reinforcements were used based on the required thickness of the laminates.

Thermoplastic hybrid composite laminate configurations using Elium^®^ 150 resin were manufactured at RT. After mixing the resin with hardener at a weight ratio 100:3, it was injected into the preform at full vacuum. After injection, the laminate was let to cure at RT for approximately one hour and the cured laminate was demoulded and then post-cured at 60 °C for an hour. Similarly, a heated cycle was employed for thermoset hybrid composite laminate, containing epoxy as the matrix. The mould temperature before injection was raised to 50 °C and the prepared resin by mixing it with hardener at a weight ratio of 100:35 was also kept at 50 °C. Further, the laminate was injected at this temperature and once the injection was completed, the mould temperature was increased to 110 °C and was held at this temperature for nearly 10 min. Furthermore, the mould was brought to RT and was then demoulded.

The manufactured hybrid composite configurations were cut to the required dimensions of Mode I and Mode II based on ASTM D 5528-13 [35] and JIS K7086 [36] standards, as shown in Figure 2d and Figure 2e, respectively. The manufactured laminates configurations with their fiber volume fraction (V_f_) and thickness are illustrated in Table 1. For testing of the manufactured laminate for Mode I and Mode II testing, an aluminum doubler of thickness 1.2 mm was required to be glued to increase the stiffness, which will be explained in more detail in Section 3.

## 3. Experimental Details

In the current research work, a Mode I (Double Cantilever Beam) fracture toughness test and a Mode II (End-Notched Flexure) fracture toughness test were performed. Because of the low stiffness of the thermoplastic fibers, there are chances of the composite laminate manufactured using thermoplastic reinforcement to be failed prematurely under compression failure without any crack propagation under Mode I/flexure testing [37,38]. To find the solution to this problem different methods were tried, one method is the manufacturing of a thicker sample, which will increase the stiffness of the laminate; but, for the Mode I sample, such a sample thickness with hybrid layers is difficult to manufacture, and the chances of manufacturing defects will increase, which is not ideal for fracture toughness test. Similarly, for Mode II specimens, it may cause difficulty during testing due to the requirement of a higher support span to width ratio [38]. Additionally, the bending of the thicker sample would not be as desired and which will result in incorrect fracture toughness results. Hence, to improve the bending stiffness aluminum doublers of were added to the laminate [37]. Aluminum sheets were bonded to the laminates using a control adhesive. The extra factors influenced by the doubler plates are also taken into consideration and the equations were modified [37]. With the addition of the doubler plate, the specimen will no longer be homogenous, and the critical change will be the increase in EI, and the bending stiffness. Furthermore, the addition of doubler plates alters the compliance of the composite specimen and thus the data reduction formulae will be revised. The modified equations considering the addition of the doubler for the bending stiffness were used [37].

### 3.1. Double Cantilever Beam/Mode I Fracture Toughness Test

**Figure 3:**
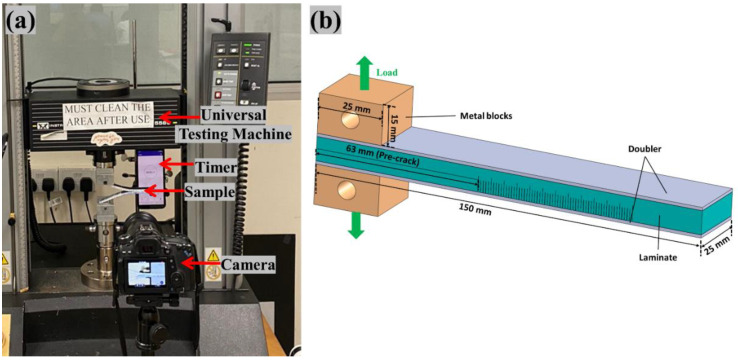
(**a**) Mode I test setup (**b**) Schematic of Mode I sample with doublers and metal block attached.

The fracture toughness test, Mode I, was carried out utilizing an Instron 5569 machine with a load cell of 50 kN, using a 3-point bending fixture following the ASTM D 5528-13 [35] standard. According to the standard, the length of the artificial delamination or crack has to be 63 mm for the Mode I test. With a cyanoacrylate adhesive, two metal blocks with through-holes were bonded to the split ends of each specimen. Figure 3b depicts the schematic of the sample dimensions. To correctly track the crack propagation of the laminate during testing, the sides of the laminates were painted with white correction fluid and the scale with each millimeter were marked as can be seen from Figure 3a.

The DCB specimen in Mode I was peeled by pulling the two metal blocks at a constant feed rate of 2 mm/min. The load and crosshead displacement was observed and noted using the data-acquisition system. To aid in the observation of crack propagation over time, a digital camera was positioned in front of the specimen (Figure 3a) [38].

**Figure 4:**
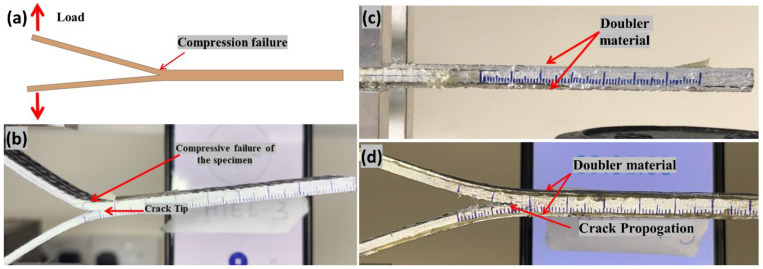
(**a**,**b**) Schematic and testing showing the compression failure of the specimen under Mode I testing without doubler respectively (**c**) Hybrid composite specimen with aluminum doubler (**d**) Mode I testing of the hybrid composite laminate with doublers.

The ASTM D 5528-13 standard was utilized to determine the inter-laminar fracture energy in Mode I [35]. Figure 4a,b depicts the premature failure of the hybrid composite laminate when tested under Mode I without doublers due to low in-plane stiffness as explained above. Figure 4c,d shows the Mode I sample glued with the doubler and when tested it undergoes the desired testing, respectively. A modified beam theory expression with correction factors for significant displacement correction and end block correction was applied. The modified equation using the doublers was shown by the equation below:

(1) $$G_{IC}=\left(\frac{L^{2}*\;{\left(a+\left(\chi *h\right)\right)}^{2}}{w*EI_{del\left(doubler\right)}}\;\right)$$

where L: load for crack growth (N), a: corresponding crack length (mm), *w*: width of the specimen (mm), h: Half-thickness of the specimen (mm), *χ* is the correction factor.

It should be noted that the χ is the correction factor, which is affected by the inclusion of the doubler, but since this factor is very small, it is neglected in the current research work.

### 3.2. End Notched Flexure/Mode II Fracture Toughness Test

**Figure 5:**
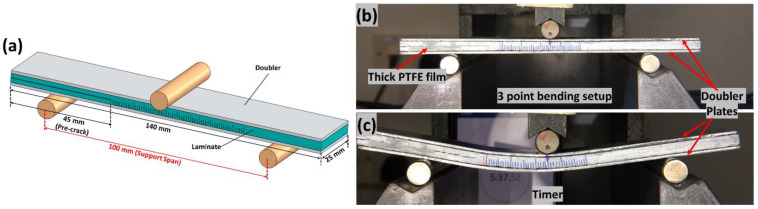
(**a**) Schematic of the Mode II tests sample with dimensions (**b**) Mode II test setup and (**c**) Specimen undergoing Mode II test.

The Mode II fracture toughness test was carried out following JIS K7086 [36] standard. Instron 5569 machine, a 3-point bending fixture, was used to perform Mode II test. Artificial delamination of 45 mm using PTFE film was created during the manufacturing for Mode II specimens. To correctly track the crack propagation of the laminate for Mode II specimens during testing, the sides of the laminates were painted with white correction fluid and the scale with each millimeter were marked as can be seen from Figure 5b. To reduce the friction between crack surfaces during Mode II test, the PTFE film of 20 μm thickness was removed, and a thick PTFE film was placed between the crack surfaces [38]. Figure 5a–c depicts the Mode II schematic, test setup, and a specimen undergoing the Mode II test, respectively. To facilitate in the observing of crack propagation with respect to time, a digital camera is positioned in front of the specimen for both the fracture toughness tests, Mode I and II [38].

The Mode-II is analyzed based on the beam theory as shown in Equation (2), with the crack length correction in accordance to JIS standard [39].

(2) $$G_{IIC}=\frac{9*L^{2}*C*a^{2}}{2*w*(\left(2*S^{3})+\left(3*a^{3}\right)\right)}$$

Compliance is given by Equation (3) [39].

(3) $$C=\frac{\left(2*L^{3}\right)+\left(3*a^{3}\right)}{8{*E}_{L}*w*h^{3}}$$

where L is load (N), a is the total crack length (mm), S is the half support span (mm), C: is the compliance (mm/N), $E_{L}$ is the longitudinal elastic modulus (MPa), and h: is half of the thickness of the laminate (mm).

**Figure 6:**
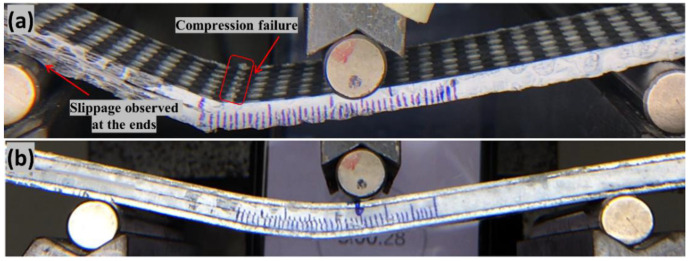
(**a**) Hybrid composite premature compression failure under mode II testing without doubler (**b**) Hybrid composite specimen under Mode II with doubler.

Based on the beam theory, the original equation for Mode-II ILFT is assessed as shown in Equation (2), JIS standard [39]. Homogeneous stiffness of the laminate is assumed in Equation (2) and therefore this equation is required to be modified to include the doubler parameter to avoid the premature compression failure (refer Figure 6a,b), which is explained in detail in our recently published work [38].

For each wholly thermoplastic composite configuration, a minimum of three specimens were tested under Mode II testing to obtain better repeatability of the result. During testing, a support span of 100 mm was used and the tensile modulus ($E_{L}$) of the composites were calculated following the ASTM D3039 [40] standard. The calculated average tensile modulus of Carbon_UHMWPP/Elium^®^ and Carbon_UHMWPE/Elium^®^ composites are 17.96 GPa and 27 GPa, respectively. Additionally, for Carbon_UHMWPP/Epoxy and Carbon_UHMWPE/Epoxy composites are 18.95 GPa and 27.94 GPa, respectively. A scanning electron microscope (SEM) was further used on the failed samples tested under Mode I and II loading to perform a detailed surface morphological study.

## 4. Results and Discussions

The test results of various mechanical characterization tests are discussed in this section. A comparative study on the performance of thermoplastic composites against thermosetting composites is also performed. In addition, the various failure mechanisms of the tested composites were carried out through a comprehensive microscopic study.

### 4.1. Double Cantilever Beam/MODE I Test

#### 4.1.1. Load vs. Displacement Characteristics

**Figure 7:**
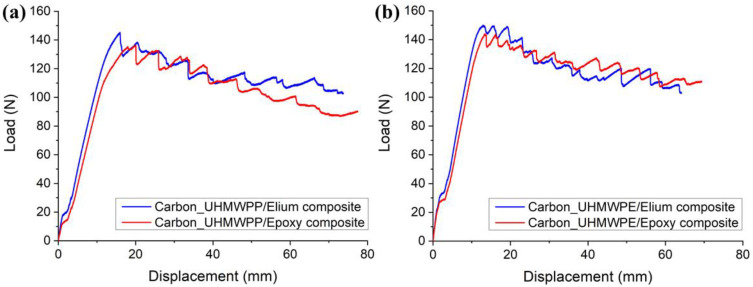
Load vs. displacement graphs for (**a**) Hybrid Carbon_UHMWPP (**b**) Hybrid Carbon_UHMWPE composite configurations.

The load vs. displacement graphs for Carbon_UHMWPP and Carbon_UHMWPE reinforced Elium^®^ and Epoxy composites are shown in Figure 7a,b, respectively. A minor non-linearity is noticed prior to the fracture propagation, following which the specimen exhibited acceptable linear behavior. The load-displacement curve of the hybrid fiber reinforced composite also shows the stick-slip behavior connected with unstable crack jumps in the weaving structure [3]. As shown in Figure 7a, peak load to failure is higher for Carbon_UHMWPP/Elium^®^ composites when compared to Carbon_UHMWPP/Epoxy composites. This could be attributable to the better fiber/matrix bonding in thermoplastic composites. Whereas Carbon_UHMWPE/Elium^®^ and Carbon_UHMWPE/Epoxy composites have a lesser difference for the peak load to failure (refer Figure 7b). Some of the load reductions and Mode I fracture resistance development with increasing crack length could generally be due to secondary energy-dissipation processes such as tow rupture and/or de-bonding occurrences [23,41,42].

**Figure 8:**
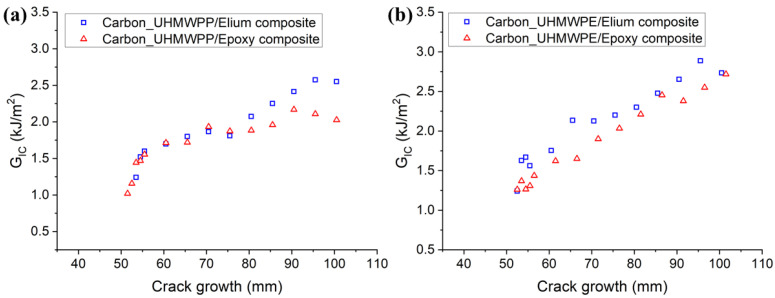
Resistance graphs for (**a**) Hybrid Carbon_UHMWPP (**b**) Hybrid Carbon_UHMWPE composite configurations.

**Figure 9:**
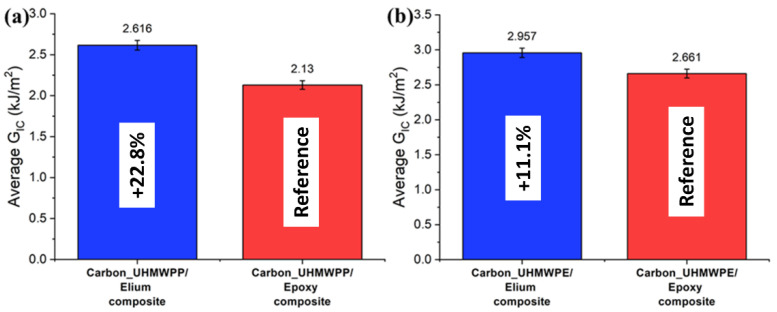
Average critical energy release rates for (**a**) Carbon_UHMWPP (**b**) Carbon_UHMWPE composites configurations.

The resistance curves (R-curves) of Carbon_UHMWPP and Carbon_UHMWPE reinforced Elium^®^ and Epoxy composites are depicted in Figure 8a,b, respectively. The hybridization of UHMWPP and UHMWPE fibers with carbon fiber results in a significant rise in G_IC_ values. Due to the disparity in carbon and thermoplastic yarn diameter and geometries, a resin-rich zone is created at hybrid interfaces, which in turn promotes a cohesive failure at the hybrid interfaces [3]. Figure 9a,b shows the average mode I fracture toughness values of various hybrid composites. For Carbon_UHMWPP/Elium^®^ composite has a G_IC_ value of 2.616 kJ/m^2^, which is 22.81% higher than Carbon_UHMWPP/Epoxy composite. However, Carbon_UHMWPE/Elium^®^ and Carbon_UHMWPE/Epoxy have G_IC_ values of 2.957 kJ/m^2^ and 2.661 kJ/m^2^, respectively (11.11% for Elium^®^ based hybrid composite).

#### 4.1.2. Failure Mechanisms

**Figure 10:**
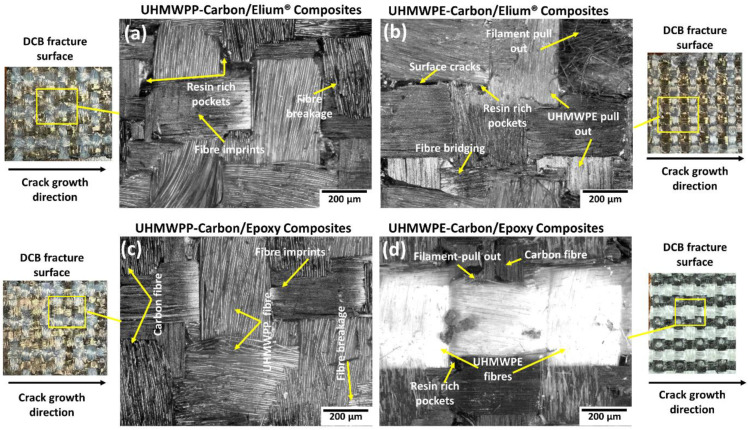
Micrographic images of the failed surfaces of (**a**) Carbon_UHMWPP/Elium^®^ (**b**) Carbon_UHMWPE/Elium^®^ (**c**) Carbon_UHMWPP/Epoxy and (**d**) Carbon_UHMWPE/Epoxy reinforced composite configurations under Mode I loading.

All the hybrid laminated composite configurations have shown stick-slip characteristics (refer Figure 7a,b) which is a very well-known phenomenon occurring particularly for the composites with woven fabric due to its weave architecture [3,4]. There is more resistance to crack propagation as the crack front is typically discontinuous and jumps in between the fiber tows in longitudinal and transverse directions. The interfaces of the hybrid composite configuration with both the matrix systems have the same amount of UHMWPP or UHMWPE fibers and carbon fibers. Figure 10a–d shows the microscopic images of the different laminate configurations. For Carbon_UHMWPP and Carbon_UHMWPE reinforced Elium^®^ composites (refer Figure 10a,b), extensive fiber bridging, and fiber breakage and the pull-out phenomenon were observed, which results in significantly resisting the crack propagation. It is evident for all the laminate configurations that the layers with the interweaving of UHMWPE and UHMWPP with carbon fibers have formed a tortuous interface as there is a presence, are matrix/resin-rich sites, as well as the varied ranges of yarn sizes as well as differences in the adhesion characteristic of these reinforcements with acrylic thermoplastic and thermosetting epoxy resins (refer Figure 10a,b,d). This is also one of the reasons for the laminated configurations showing very strong stick-slip characteristics. The fiber failure sites at the resin-rich areas are observed along with the pull out of the UHMWPE yarns with the epoxy matrices highlighting the poor adhesion resins (refer Figure 10d).

**Figure 11:**
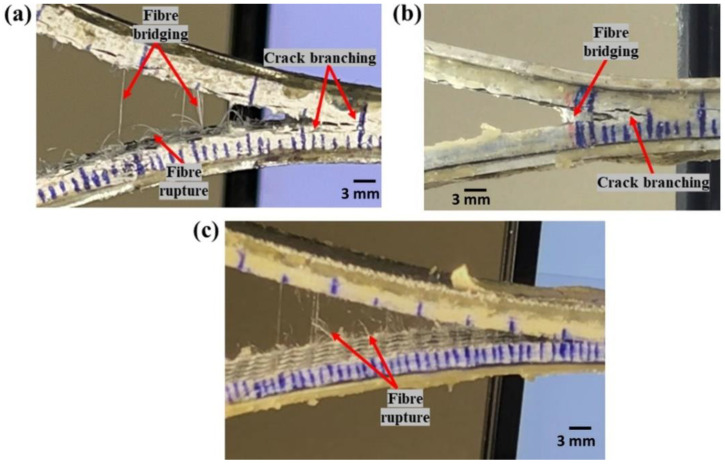
Macro-photographs of side surfaces of (**a**,**b**) Carbon_UHMWPP and (**c**) Carbon_UHMWPE reinforced thermoplastic composites.

To better understand the failure mechanisms, several macro and micro photographs of the side surface of the specimen were taken. Figure 11a–c shows the macro-photographs of hybrid fiber reinforced thermoplastic composites, which highlight the features, such as fiber bridging and pull out, formation of multiple cracks sites, crack branching, and others that are important crack arresting mechanisms. The resistance to fracture growth is also aided by localized filament pull-out and splitting of thermoplastic yarns [3].

**Figure 12:**
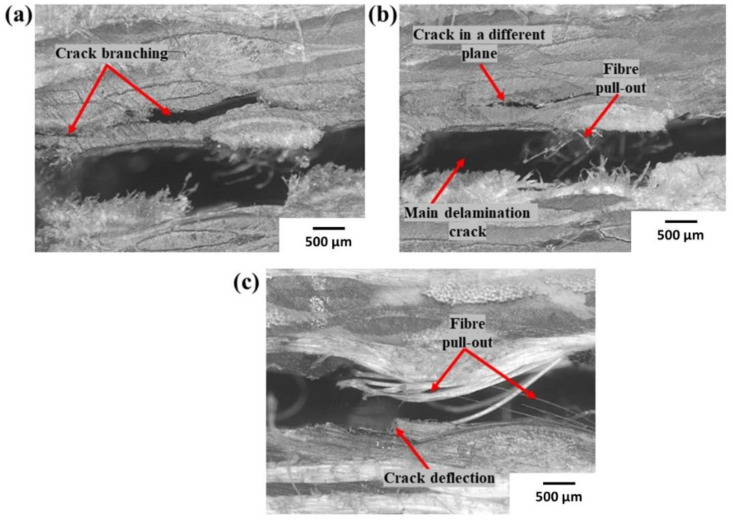
Micro-photographs of side surfaces of (**a**,**b**) Carbon_UHMWPP and (**c**) Carbon_UHMWPE reinforced thermoplastic composites.

Microphotographs of Carbon_UHMWPP and Carbon_UHMWPE reinforced thermoplastic composites with multiple cracks, fiber pull-outs are described in Figure 12a–c, respectively. The typical features shown by Carbon_UHMWPP reinforced composites included multiple cracks and crack deflection along with fiber pull-outs and bridging. While Carbon_UHMWPE reinforced composites exhibit only moderate fiber pull-out and rupture due to the poor adhesion characteristics of the UHMWPE fabrics with both the thermoplastic and thermoset variants.

The initial crack deflection sites and the subsequent crack branching significantly contribute to improving the delamination resistance of the hybrid fabric with acrylic Elium^®^ resin [9,20]. The crack advancement in the interlayers is highly resisted due to the higher fracture toughness of Elium^®^ resin (0.5 kJ/m^2^) compared to thermoset epoxy resin (0.2 kJ/m^2^) [9,20,23].

**Figure 13:**
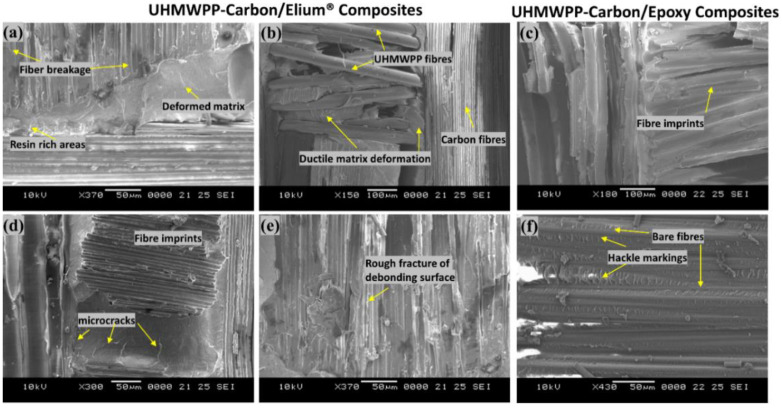
Surface morphological images of fractured surfaces of composites using Scanning Electron Microscopic approach of (**a**,**b**,**d**,**e**) Carbon_UHMWPP/ Elium^®^ (**c**,**f**) Carbon_UHMWPP/Epoxy composites under Mode I loading.

**Figure 14:**
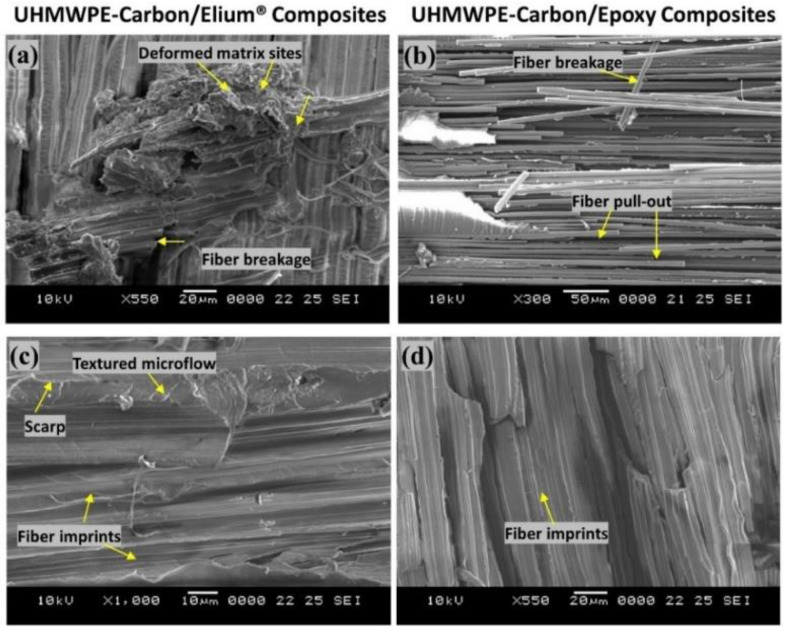
Surface morphological images of fractured surfaces of composites using Scanning Electron Microscopic approach of (**a**,**c**) Carbon_UHMWPE/Elium^®^ (**b**,**d**) Carbon_UHMWPE/Epoxy composite under Mode I loading.

To further understand the failure processes, a scanning electron microscopic analysis was used to examine the tested specimens in detail. The different failure modes of hybrid fiber reinforced composites are depicted in Figure 13a–f and Figure 14a–d. Hybrid fiber reinforced thermoplastic composites shows severe plastic deformation of the matrix, rough fracture residue and micro-cracks on the de-bonding surface (refer Figure 13a,b,d,e and Figure 14a), which in turn confirms better fiber/matrix bonding. Figure 13a shows a resin-rich region at the hybrid interface of the Elium^®^ composite, indicating the possibility of a cohesive failure. Localized surface fractures are also observed immediately after crack initiation sites, which are prevented by fiber pull-outs, bridging, and a strong fiber-matrix bonding (refer Figure 13a,d and Figure 14a,c).

Sharp step features, such as scarp (refer Figure 14a) and the textured microflow sites, are also observed for the acrylic-based composites (refer Figure 14c). While hybrid fiber reinforced epoxy composites exhibit hackle markings and clean fiber fracture with smooth surface and pull-outs, which is a typical characteristic of a brittle thermoset matrix system [9,23] as shown in Figure 13f and Figure 14b Fiber imprints were noticed for all the laminated composite configurations (refer Figure 13c,d and Figure 14c,d) with matching surfaces being exposed with one leaving with a significant amount of fibers while the other has it imprints [43].

### 4.2. End Notched Flexure/MODE II Test

#### 4.2.1. Load vs. Displacement Characteristics

**Figure 15:**
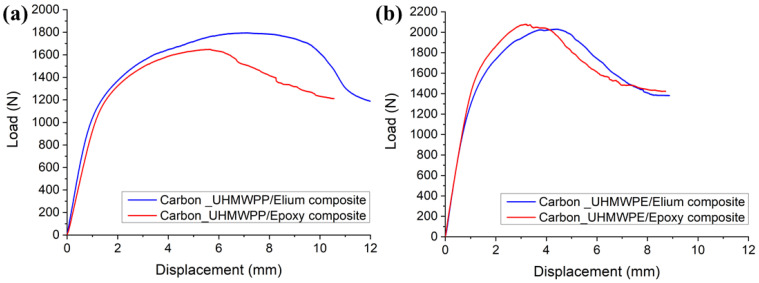
Load vs. Displacement graphs for (**a**) Carbon_UHMWPP (**b**) Carbon_UHMWPE fiber reinforced composite configurations.

The load vs. displacement graphs for Carbon_UHMWPP and Carbon_UHMWPE reinforced Elium^®^ and Epoxy composites under Mode II loading are shown in Figure 15a,b, respectively.

The load vs. displacement graphs of hybrid fiber reinforced composites exhibit a linear growth followed by a non-linear portion, which is more noticeable in the case of thermoplastic composites, before the onset of crack propagation. However, the non-linearity observed in Elium^®^ composites is indicative of the thermoplastic resin plastic deformation which delays the unstable crack propagation to a greater displacement value [11], as shown in Figure 15a,b. The highest peak load is found for Carbon_UHMWPP reinforced composites using Elium^®^ as the matrix, suggesting an 8.88% increase in load-bearing capability above the baseline of Carbon_UHMWPP reinforced epoxy composite. However, Carbon_UHMWPE/Epoxy composite has a higher peak load of 2.079 kN, which is 2.36% greater than that of Carbon_UHMWPE/Elium^®^.

**Figure 16:**
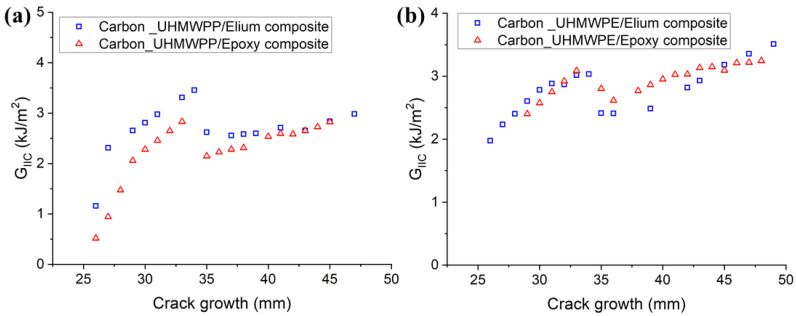
R-curves for (**a**) Carbon_UHMWPP (**b**) Carbon_UHMWPE composites configurations.

Figure 16a,b depict the resistance curves for Carbon_UHMWPP and Carbon_UHMWPE reinforced Elium^®^ and Epoxy composites, respectively. The crack growth is found to be relatively more unstable for Elium^®^ composites. A considerable increase in the fracture toughness values is observed for based hybrid fiber composites. This could be due to the resin pockets created by the difference in the carbon and thermoplastic fiber diameters and the tortuous crack propagation [4].

**Figure 17:**
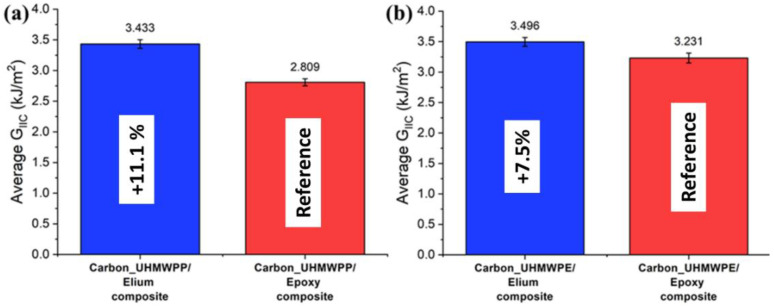
Average critical energy release rates for (**a**) Carbon_UHMWPP (**b**) Carbon_UHMWPE composites configurations.

The results of all the laminated configurations compared to the Mode I test results are more dispersed due to the complexity of the toughening mechanisms for hybrid thermoplastic composites. The non-linearity as observed from the R-curves of the hybrid composites could be attributed to the moderate to poor bonding of the thermoplastic yarns with acrylic Elium^®^ and Epoxy resin, respectively, as well as the significantly lower transverse and shear properties of UHMWPE and UHMWPP fibers, and the same has been reported in a research performed by Zhou et al., while investigating the Mode II performance of hybrid carbon-UHMWPE/epoxy composite [4]. The average mode II fracture toughness values of hybrid fiber reinforced composites are illustrated in Figure 17a,b, respectively. The G_IIC_ value of the Carbon_UHMWPP/Elium^®^ composite is 3.433 kJ/m^2^ which is 22.21% higher than the G_IIC_ value of the Carbon_UHMWPP/Epoxy composite. However, Carbon_UHMWPE/Elium^®^ and Carbon_UHMWPE/Epoxy have G_IIC_ values of 3.496 kJ/m^2^ and 3.231 kJ/m^2^, respectively. The increase in the Mode II fracture toughness for the acrylic-based hybrid composites is attributed to the difference in their chemical structures with Elium^®^ absorbing more energy as there is comparatively a much larger free volume between the polymeric chains and hence leads to additional plastic deformation absorption linked to the crack propagation, while the thermoset epoxy-based composites have cross-linked structure inducing brittle characteristics leading to lower fracture toughness [9,11,44]. The fracture toughness for hybrid composites is relatively higher than the pure carbon variants due to the extra resistance arising from the frictional sliding of the expanding contact area of the woven fabric of the failure surfaces [3,4,9]. Thermoplastic fabrics are known to improve the friction between the surfaces of the fracture zones in contrast with the smooth surface in the case of conventional carbon fabrics [3,4].

#### 4.2.2. Failure Mechanisms

**Figure 18:**
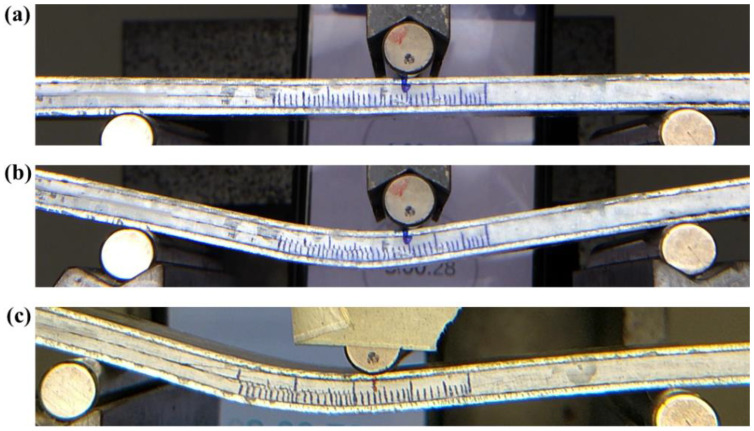
Macro-photographs of (**a**) Carbon_UHMWPE/Elium^®^ composite at the start of the test (t = 0 s) (**b**) Carbon_UHMWPE/Elium^®^ composite between testing (t = 480 s) (**c**) Carbon_UHMWPE/Epoxy composite between testing (t = 480 s).

Macro-photographs of crack growth of Carbon_UHMWPE reinforced composites taken during the Mode II test are shown in Figure 18a–c. Figure 18a,b represent Carbon_UHMWPE/Elium^®^ sample at the beginning of the test and at a test duration of t = 480 s, respectively, whereas Figure 18c represents the Carbon_UHMWPE/Epoxy sample at a test duration of t = 480 s. A crack propagation of 18 mm and 23 mm can be observed in the same time duration for Carbon_UHMWPE/Elium^®^ and Carbon_UHMWPE/Epoxy, respectively; thus, demonstrating the higher resistance to crack propagation offered by Elium^®^ composites even when they are reinforced with the same hybrid fibers.

**Figure 19:**
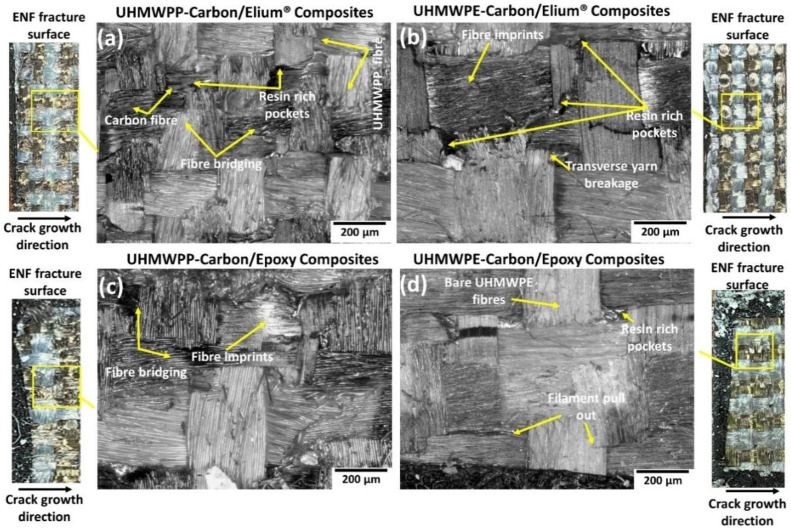
Micrographic images of the failed surfaces of (**a**) Carbon_UHMWPP/Elium^®^ (**b**) Carbon_UHMWPE/Elium^®^ (**c**) Carbon_UHMWPP/Epoxy and (**d**) Carbon_UHMWPE/Epoxy reinforced composite configurations under Mode II loading.

The microphotographs as seen from Figure 19a,b shows significant fiber bridging for the acrylic-based hybrid composite configurations. The fabric yarns and filaments are seen to be pulled out being adjoined to the crack surfaces along with the transverse yarn damage and signals that at the delamination front there must have been a formation of bridging zones which is an important crack resistance mechanism (refer Figure 19a,b). Due to mismatches in the yarn sizes of the UHMWPE or UHMWPP with carbon, large resin pockets or resin-rich areas (refer Figure 19a,b,d) are introduced which is an important driver to improving the fracture toughness by aiding in an increased fracture process zone. For the epoxy-based hybrid composites, the fiber dominating crack enhancing mechanisms along with imprints remains intact but the presence of bare fibers deprived of the resin signals a poor adhesion of the thermoplastic fabrics with epoxy resin similar to the earlier reported mechanisms for Mode I results (refer Figure 19c,d).

**Figure 20:**
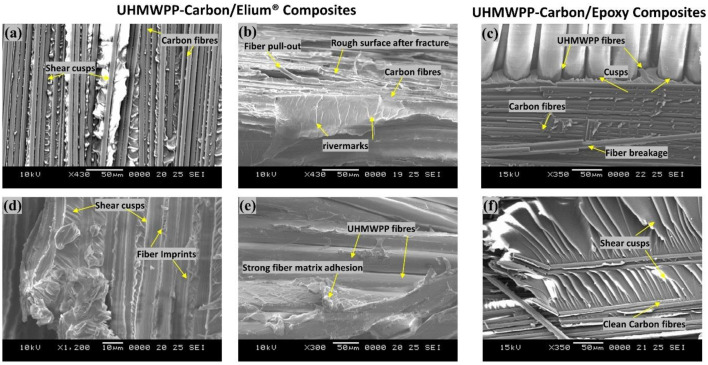
Surface morphological images of fractured surfaces of composites using Scanning Electron Microscopic approach of (**a**,**b**,**d**,**e**) Carbon_UHMWPP/Elium^®^ composite (**c**,**f**) Carbon_UHMWPP/Epoxy composite under Mode II loading.

**Figure 21:**
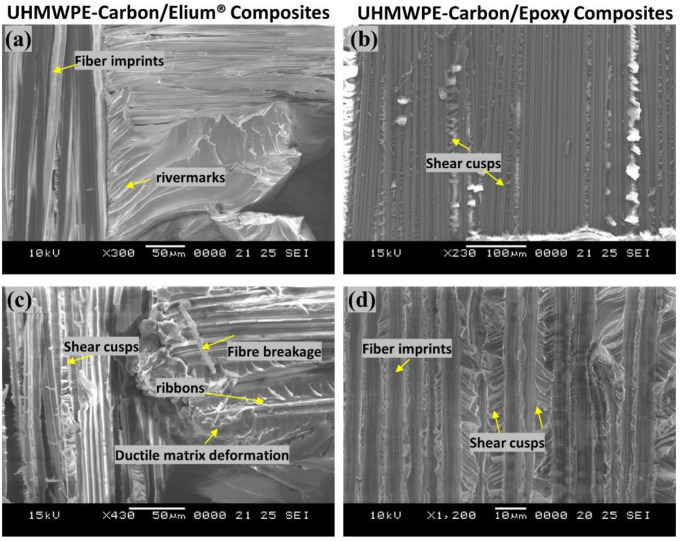
Surface morphological images of fractured surfaces of composites using Scanning Electron Microscopic approach of (**a**,**c**) Carbon_UHMWPE/Elium^®^ composite (**b**,**d**) Carbon_UHMWPE/Epoxy composite under Mode II loading.

For a better comprehension of the failure mechanisms of the various hybrid composite configurations, an elaborate Scanning Electron Microscopy study was conducted, as illustrated in Figure 20a–f and Figure 21a–d. Figure 20a–f and Figure 21a–d depict the presence of shear cusps, river line marking converging to form scarps, fiber imprints, and fiber pull-out, which are all common Mode II failure features. Strong fiber/matrix adhesion of Elium^®^ composites is observed in Figure 20e and Figure 21c. Figure 21c depicts the severe ductile plastic deformation underwent by the Elium^®^ matrix. Figure 20c,f and Figure 21b,d shows the various failure modes observed in Epoxy composites including the shear cusps and fiber imprints. The smooth de-bonding surface illustrated the bare fiber (refer Figure 20f) which substantiates the poor fiber/matrix adhesion of the thermosetting composite. One can observe the presence of the bare fibers resulting from the shear failure of the matrix under consideration but on the contrary, the surfaces were found to be significantly rougher in acrylic Elium^®^ based hybrid composites owing to significant yarn splitting of the thermoplastic fibers. Thermoplastic fibers, such as UHMWPE and UHMWPP are chemically inert and tend to form weaker bonds with the thermosetting epoxy as well as acrylic Elium^®^ resin in the current investigation [3,4]. The fracture surfaces are also found to be partially covered with a very thin epoxy or acrylic resin layer along with the fiber imprints of the fibers, which were debonded from the mating layer and confirm the delamination growth or propagation on the bottom fiber/matrix interface (refer Figure 20b,c,e,f and Figure 21a–d). At a higher magnification, thermoplastic fibers are observed to be pulled out and are nicely correlated to the macro photographic fiber bridging aspects, which are discussed earlier (refer Figure 20b,c and Figure 21c).

## 5. Conclusions

In the current research, hybrid fiber reinforced Elium*^®^* composites are manufactured using a vacuum-assisted resin infusion (VARI) process, and their fracture toughness attributes are studied under Mode I and II loading. The details of the experimental study using the doubler methodology are also presented to test the hybrid laminated composite configurations. The failure mechanisms of the composites under each of the above-mentioned loading scenarios are understood by the detailed microscopic investigation to comprehend the bonding efficacy of thermoplastic Elium*^®^* resin with the hybrid thermoplastic fibers along with the baseline comparison carried out with the composites manufactured using thermosetting epoxy matrix. Important findings are summarized below:

The stiffness of the composite laminates is modified by including aluminum doublers and the Mode I and II tests were successfully carried out for all the hybrid laminated composite configurations by eradicating the concerns of large displacement and compression failure during the tests.

Hybrid Carbon_UHMWPP/Elium^®^ composite has shown 22.81% higher G_IC_ and 22.2% higher G_IIC_ than Carbon_UHMWPP/Epoxy composite. While the Carbon_UHMWPE/Elium*^®^* has shown an 11.11% higher G_IC_ and a 7.58% higher G_IIC_ than Carbon_UHMWPE/Epoxy composite.

The initial crack deflection sites and the subsequent crack branching contribute significantly to improving the delamination resistance of the hybrid fabric with acrylic Elium*^®^* resin under the Mode I loading scenario. Hybrid fiber reinforced thermoplastic composites shows severe plastic deformation of the matrix, rough fracture residue, and micro-cracks on the de-bonding surface, which resulted in the improved fracture toughness for the hybrid thermoplastic-based composites while the hybrid fiber reinforced epoxy composites exhibit hackle markings and clean fiber fracture with smooth surface and pull-out.

In general, hybrid fiber architecture is also found to be contributing significantly to increasing the overall fracture toughness as it induced the tortuous crack path due to the mismatch of the fiber yarns and the generation of resin-rich sites which has a major positive say on the fracture toughness attributes.

The development of hybrid fiber reinforced thermoplastic composites and their acceptable bonding with the thermoplastic acrylic resin could be an excellent alternative to the conventionally used thermosetting material systems owing to their ease of manufacturing at room temperature along with the typical thermoplastic advantages they will offer in terms of impact, damping, recyclability, and many others which could have tremendous application in automotive, sporting equipment, and protective gear and ballistic applications.

## Figures and Tables

**Table 1:** Manufactured laminates configurations with their thickness, fiber volume fraction, and nomenclature used in current research.

Reinforcement System	Resin System	Layers	Fiber Volume Fraction (%)	Thickness (mm)	Terminology
Carbon-UHMWPP	WAM Epoxy	12	48.7	7.3	Carbon_UHMWPP/Epoxy
	Elium^®^		49.4	7.2	Carbon_UHMWPP/Elium^®^
Carbon-UHMWPE	WAM Epoxy	14	49.7	7.3	Carbon_UHMWPE/Epoxy
	Elium^®^		50.8	7.2	Carbon_UHMWPE/Elium^®^

Note: Thickness indicated in the table is the total laminate thickness inclusive of Al doubler.

## Data Availability

The raw data required to reproduce these findings cannot be shared at this time as the data also forms part of an ongoing study.
